# Commencing Hernia

**Published:** 1885-01

**Authors:** J. Matthews Duncan


### Commencing Hernia.

All pains about the external inguinal opening are not neuralgic, nor inflammatory. I remember well the case of a sensitive young lady whose pains had been very puzzling to her physician, who had reasons for avoiding strict local inquiries. She was indeed sent to me to have these inquiries made, because much and varied treatment by medicines and rest had failed to give any relief. The pain was confined to the region of the femoral opening on one side; rest removed it, exertion induced it, coughing made it worse; and a strong impulse was felt in the part by the hand applied when a deep cough was made. Knowledge of the seat and cause of the pain was nearly enough for its cure, and complete relief was got by wearing a truss. Cases of a like kind are not very rare.—*J. Matthews Duncan in Med. Times and Gazette.*

---

THE death-rate of Russia is the highest in Europe. This is attributed to the paucity of medical men and the habits of the rural population. According to late returns the average duration of life is only 26 years, and the mortality among infants is frightful. More than 60 per cent. of infants die before they reach their fifth year, and nearly 2,000,000 children perish every year. Of 8,000,000 boys, only 3,770,000 attain the age of military service—that is to say, their twenty-fifth year, and of these at least 1,000,000 are found, by reason of shortness of stature and weakness of body, to be unfit for military duties.—*Ex.*

---

THE chemist of the Sanitary Bureau has examined several hundred specimens of mustard, collected under the direction of Dr. Edson, and has submitted the results of his examination to the New York Board of Health. Three specimens contained flour and were colored by the addition of naphthol yellow. Ten other specimens contained flour or *terra alba*, and in some cases there was only 35 per cent. of mustard. Naphthol yellow is an irritant poison.

